# Are we ever too old? Characteristics and outcome of octogenarians admitted to a medical intensive care unit

**DOI:** 10.1097/MD.0000000000007776

**Published:** 2017-09-15

**Authors:** Johanna Maria Muessig, Maryna Masyuk, Amir Movahed Nia, Marcus Franz, Bjoern Kabisch, Malte Kelm, Christian Jung

**Affiliations:** a Division of Cardiology, Pulmonology, and Vascular Medicine, Medical Faculty, University Hospital Düsseldorf, Heinrich-Heine-University, Düsseldorf; b Department of Cardiology, Clinic of Internal Medicine I, Medical Faculty, Jena University Hospital, Friedrich-Schiller-University, Jena, Germany.

**Keywords:** elderly, intensive care, mortality, resource consumption, short- and long-term outcomes

## Abstract

The aging population increases the demand of intensive care unit (ICU) treatments. However, the availability of ICU beds is limited. Thus, ICU admission of octogenarians is considered controversial. The population above 80 years is a very heterogeneous group though, and age alone might not be the best predictor. Aim of this study was to analyze resource consumption and outcome of octogenarians admitted to a medical ICU to identify reliable survival predictors in a senescent society.

This retrospective observational study analyzes 930 octogenarians and 5732 younger patients admitted to a medical ICU. Admission diagnosis, APACHE II and SAPS II scores, use of ICU resources, and mortality were recorded. Long-term mortality was analyzed using Kaplan-Meier survival curves and multivariate cox regression analysis.

Patients ≥80 years old had higher SAPS II (43 vs 38, *P* < .001) and APACHE II (23 vs 21, *P* = .001) scores. Consumption of ICU resources by octogenarians was lower in terms of length of stay, mechanical ventilation, and renal replacement therapy. Among octogenarians, ICU survivors got less mechanical ventilation or renal replacement therapy than nonsurvivors. Intra-ICU mortality in the very old was higher (19% vs 12%, *P* < .001) and long-term survival was lower (HR 1.76, *P* < .001). Multivariate cox regression analysis of octogenarians revealed that admission diagnosis of myocardial infarction (HR 1.713, *P* = .023), age (1.08, *P* = .002), and SAPS II score (HR 1.02, 95%, *P* = .01) were independent risk factors, whereas admission diagnoses monitoring post coronary intervention (HR .253, *P* = .002) and cardiac arrhythmia (HR .534, *P* = .032) had a substantially reduced mortality risk.

Octogenarians show a higher intra-ICU and long-term mortality than younger patients. Still, they show a considerable life expectancy after ICU admission even though they get less invasive care than younger patients. Furthermore, some admission diagnoses like myocardial infarction, cardiac arrhythmia and monitoring post cardiac intervention are much stronger predictors for long-term survival than age or SAPS II score in the very old.

## 1 Background

Life expectancy is growing all over the world.^[1]^ During the last 20 years, the number of very old (≥80 years) people increased considerably all over the world but especially in the developed countries. In the next decades, aging of the population is predicted to further increase: globally, the number of persons aged 80 years or older is expected to more than triple by 2050 making the very old the fastest growing group of the worlds’ population.^[1]^ The depicted development increases the demand of intensive care unit (ICU) treatments.^[2]^ Even though the group of the very old contributed to only 4% of the population in developed countries in 2010,^[1]^ they accounted for more than 10% of ICU admissions in the Western world.^[3–7]^ However, the costs for intensive care are high and the availability of ICU beds is limited. Previous studies have shown a worse outcome for ICU patients aged ≥80 years compared with younger patients.^[8,9]^ Thus, ICU admission of octogenarians is discussed controversially.^[10]^ However, there is a high mortality among very old patients that are appropriately referred for intensive care but whom ICU admission was denied.^[11,12]^ The population above 80 years represents a very heterogeneous collective. Even though age is shown to be an independent risk factor for hospital and ICU mortality,^[8,13,14]^ age alone might not be the best predictor. Furthermore, commonly used scoring systems that were developed to quantify the severity of illness and the likelihood of survival such as SAPS II and APACHE II score were established and validated for a general ICU population. Thus, there is an ongoing debate whether these scores sufficiently depict the situation of distinct subpopulations of ICU patients such as octogenarians.^[15,16]^ Several studies have shown correlations of the cause of ICU admission and long-term survival: very old patients admitted because of medical diagnoses were shown to have a worse outcome compared to patients admitted for elective or unscheduled surgery.^[8,14,17]^ However, to the best of our knowledge there is no study focusing on the impact of different medical admission diagnoses on intra-ICU and long-term mortality of very old medical ICU patients. The aim of this study was to analyze resource consumption and outcome of octogenarians admitted to a medical ICU to identify reliable survival predictors in a senescent society.

## 2 Methods

### 2.1 Study design and study population

This single center retrospective observational study analyzed resource consumption and outcome of octogenarians admitted to a medical ICU in a German tertiary care hospital in order to identify reliable survival predictors in a senescent society. The study was based on a database of 6662 ICU patients admitted consecutively to the medical ICU at the Jena University Hospital between 2006 and 2009. Thus, the enrollment criterion for this study was the admission to the aforementioned ICU. There were no exclusion criteria for the patients that qualified for enrollment. Patients were divided into 2 groups: patients aged ≥80 (930 patients) years and patients aged <80 years (5732 patients).

Follow-up of patients was performed between May 2013 and November 2013. The primary endpoint of the study was death of any cause. Data on mortality were achieved by review of electronic in-hospital medical records or phone interviews.

The study was approved by the local ethics committee of the Medical Faculty of the Friedrich-Schiller-University Jena.

### 2.2 Calculation of SAPS II and APACHE II scores

Initial Simplified Acute Physiology Score II (SAPS II) and Acute Physiology and Chronic Health Evaluation II (APACHE II) scores were calculated by the treating physician within 24 hours after admission as reported before.^[18,19]^

### 2.3 Calculation of ICU length of stay and short-term outcome

For calculation of ICU length of stay and time of mechanical ventilation, noninvasive ventilation, and renal replacement therapy for all patients were included. For patients that died in the ICU, the hours from admission to the ICU or from the start of the mechanical or noninvasive ventilation or renal replacement therapy, respectively, until the end of the therapy (due to medical decisions or death) were accounted.

### 2.4 Statistical analyses

Statistical analyses were performed using SPSS (IBM Corp Released 2013. IBM SPSS Statistics for Windows, Version 22.0. Armonk, NY: IBM Corp). For continuous variables, normally distributed data is given in mean ± standard deviation and compared by Student *t* test. Nonnormally distributed data is shown as median with interquartile range and compared by Mann-Whitney *U* test. Categorical variables were described by counts and percentages. Differences between groups were calculated by chi-square test. Kaplan-Meier curve and log-rank test were used to depict survival data and cox regression analysis was used to compare survival data. *P* values ≤.05 were considered significant.

## 3 Results

Using a large ICU database, a total number of 6662 patients consecutively admitted to a medical ICU in a German tertiary care hospital were retrospectively investigated. Among this cohort, there were 930 patients aged ≥80 years (defined as very old patients) representing 14% of all admissions. The patients younger than 80 years served as controls. Mean follow-up time was 2141 ± 24 days. Patient characteristics are shown in Table 1.

**Table 1:**
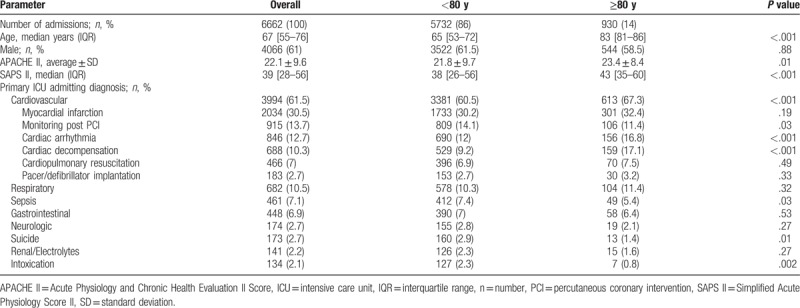
Characteristics of the study population.

The very old patients were significantly older (median 83 years [81–86] IQR vs 65 [53–72], *P* < .001) and more diseased than the younger patients reflected by both higher SAPS II (median 43 [35–60] IQR vs 38 [26–56], *P* < .001) and APACHE II (median 23 [17–29] vs 21 [14–29], *P* = .001) scores. Gender distribution was similar in both groups (544 [58.5%] male vs 3522 [61.5%] male, *P* = .88) with sex-specific differences in ICU admission rates with more males than females being admitted in both groups. The leading admission diagnoses of both groups are shown in Table 1.

In accordance with their more severe diseased state, octogenarians showed a higher intra-ICU mortality compared with younger patients (173 [18.6%] vs 696 [12.1%], *P* < .001). However, they spent fewer hours at the ICU (median 31 [18–63] IQR vs 34 [19–77] IQR, *P* = .001) and got less invasive care. Thus, a smaller percentage of very old patients received renal replacement therapy (72 [7.7%] vs 676 [11.8%], *P* < .001) as shown in Table 2. Furthermore, octogenarians getting mechanical ventilation were ventilated for fewer hours than younger patients (median 18 [5–54] IQR vs 50 [11–166] IQR, *P* < .001). However, a higher percentage of very old patients got noninvasive ventilation (NIV, 184 [19.8%] vs 940 [16.4%], *P* = .01) as shown in Table 2.

**Table 2:**
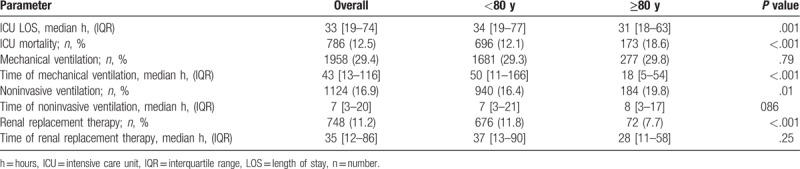
Use of ICU resources and short-term outcome.

Although we found no difference regarding age (median 83 [81–85] IQR vs 84 [81–86] IQR, *P* = .31) and sex (450 [59.4%] male vs 94 [54.3%] male, *P* = .23), ICU-survivors showed significantly lower APACHE II (mean 21.6 ± 7.7 vs 29.6 ± 7.9, *P* < .001) and SAPS II (median 40 [33–52] IQR vs 60.5 [47–75] IQR, *P* < .001) scores compared to nonsurvivors, reflecting a less severe state of disease. ICU survivors were more often admitted because of cardiovascular diseases (512 [69%] vs 101 [59.8%], *P* = .02) whereas nonsurvivors were more often admitted because of respiratory diseases (30 [17.8%] vs 74 [10.0%], *P* = .007) or sepsis (22 [13.0%] vs 27 [3.6%], *P* < .001). With regard to specific cardiovascular diseases, ICU survivors were more often admitted for monitoring post PCI (104 [13.7%] vs 2 [1.2%], *P* < .001) or after pacemaker/defibrillator implantation (30 [4%] vs 0 [0%], *P* = .003) whereas nonsurvivors were more often admitted due to cardiac decompensation (41 [23.7%] vs 118 [15.6%], *P* = .01) or cardiopulmonary resuscitation (30 [17.3%] vs 40 [5.3%], *P* < .001) as shown in Table 3.

**Table 3:**
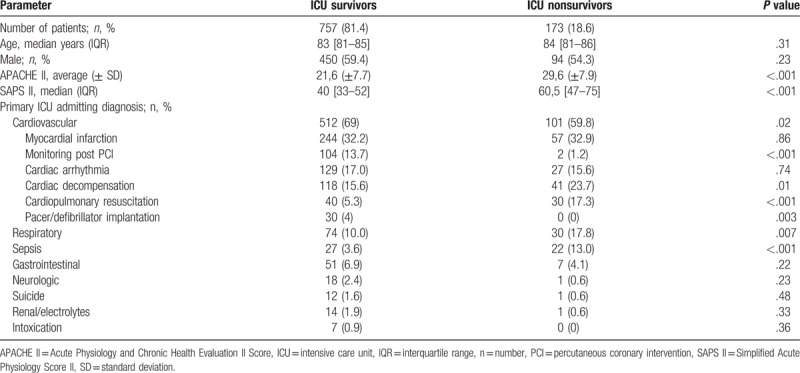
Baseline characteristics of octogenarians according to ICU survival status.

In accordance to their less diseased state, a lower percentage of ICU survivors got renal replacement therapy (35 [4.6%] vs 37 [21.4%], *P* < .001), mechanical ventilation (154 [20.3%] vs 123 [71.1%], *P* < .001), or NIV (125 [16.5%] vs 59 [34.1%], *P* < .001) compared to nonsurvivors as shown in Table 4.

**Table 4:**
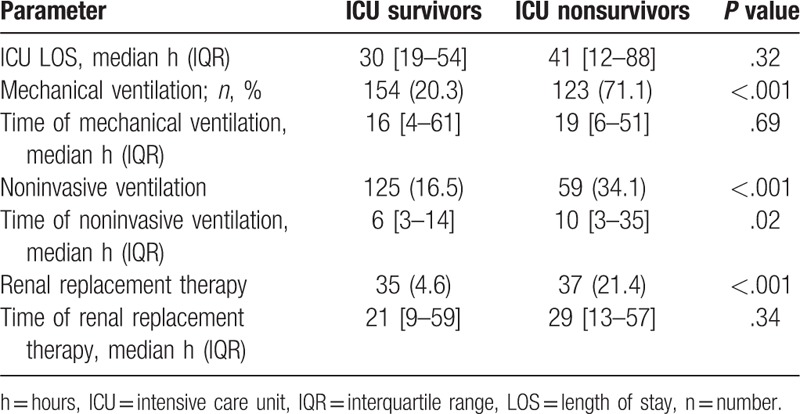
Short-term outcome and use of ICU resources of octogenarians according to ICU survival status.

**Figure 1:**
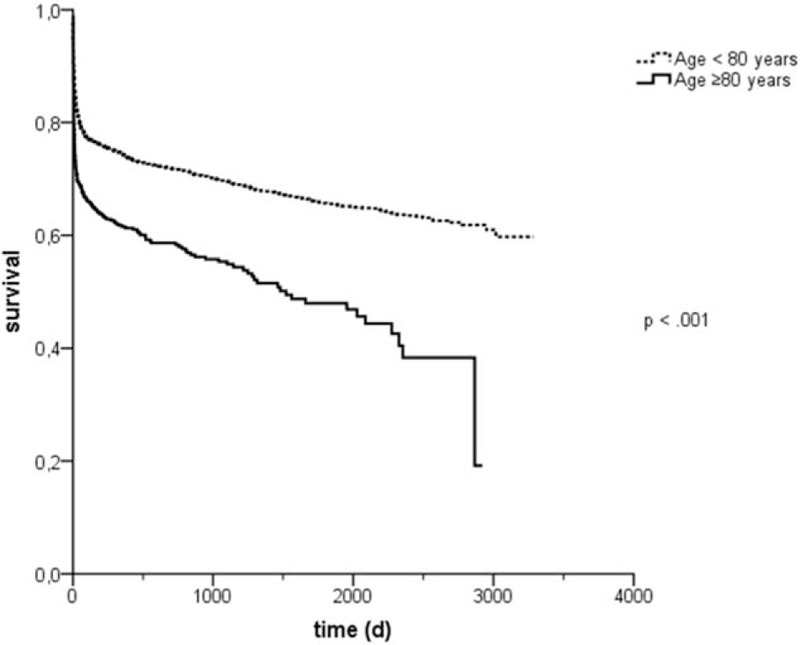
Kaplan-Meier survival curves. Age ≥80 y was associated with increased mortality in the long term (HR = 1.76, 95% CI [1560–1989], *P* < .001), with a mean survival of 1480 ± 62 d in patients ≥80 y old and 2214 ± 25 d in younger patients (*P* < .001).

To analyze the impact of age on long-term survival we compared Kaplan-Meier survival curves of octogenarians and younger patients as shown in Figure 1. Patients aged ≥80 years showed a significantly increased long-term mortality (HR = 1.76, 95% CI [1.560–1.989], *P* < .001) with a mean survival of 1480 ± 62 days compared with 2214 ± 25 days in younger patients (*P* < .001).

In multivariate cox regression analysis among the very old patients, we identified age (HR = 1.076 per year, 95% CI [1.028–1.127], *P* = .002) and SAPS II score (HR = 1.020 per point, 95% CI [1.005–1.036], *P* = .01) as well as the admission diagnosis myocardial infarction (HR = 1.713, CI [1.078–2.721], *P* = .02) as independent long-term mortality risk factors in octogenarians. The admission diagnoses cardiac arrhythmia (HR = .534, 95% CI [.301–.94], *P* = .03) and monitoring post PCI (HR = .253, 95% CI [.106–.607], *P* = .002) were independently negatively correlated with long-term mortality as shown in Table 5.

**Table 5:**
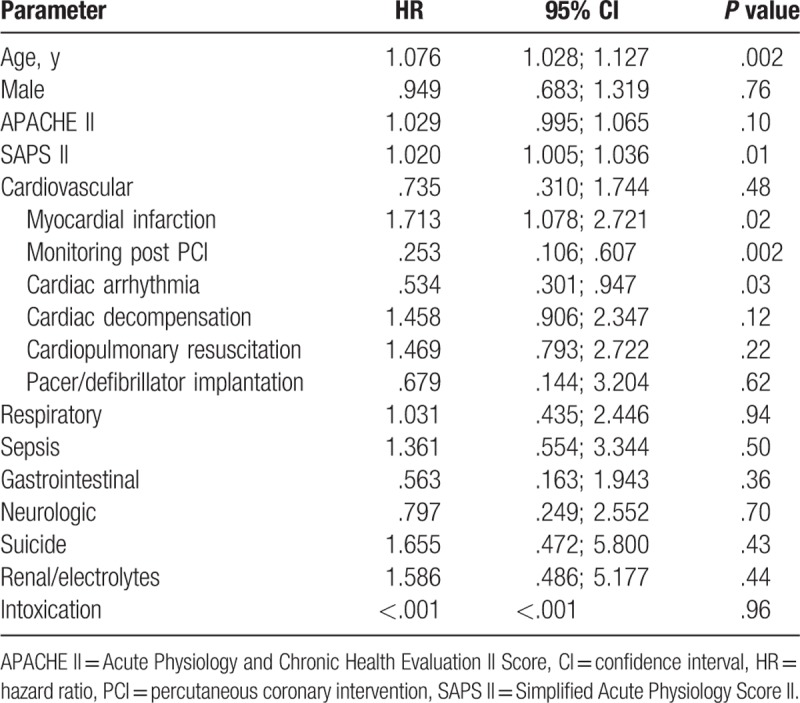
Cox regression analysis for prediction of mortality.

## 4 Discussion

This study retrospectively evaluates characteristics as well as short- and long-term outcomes of octogenarians admitted to a German medical ICU over a time period of 4 years. In our analysis old patients accounted for 14% of all ICU admissions. This goes in line with other recent studies reporting on comparable percentages of 10% to 18% octogenarians of all ICU admissions in Western countries.^[3–7]^ As already shown by other groups^[3]^ we could demonstrate gender specific ICU admission rates with more males admitted in both age groups. However, we cannot say whether that imbalance reflects triage decisions of the medical staff or the wish of patients and their relatives, as our ICU database does neither include data on severity of illness nor on treatment preferences of patients who were not submitted to our ICU. In accordance with previous studies our analyses revealed that very old patients got less invasive care compared to younger patients and had a shorter length of stay^[3,13,20]^ even though they showed more severe disease states compared to the younger cohort.^[3]^ Whether these differences are based on passive or active treatment limitation by the treating physicians or by the preferences of the patients or their families remains unclear. Furthermore, octogenarians showed a higher intra-ICU mortality, which is in line with prior investigations.^[3]^ Whether there is a relationship between reduced treatment modalities and higher mortality in the very old remains uncertain though.

One strength of our analysis is a relatively large cohort of more than 6000 patients with more than 900 octogenarians and a long follow-up time with an average of almost 6 years in the overall cohort, thus being one of the studies among very old patients with the longest follow-up. Our data revealed a considerable life expectancy of octogenarians admitted to our medical ICU with a median survival of more than 4 years. Hence our study population showed a higher median survival than previous studies^[17]^ probably due to a different case mix. With the VIP1 Study, the first large multinational study with a total number of 2000 to 10,000 ICU patients aged ≥80 years has been initiated. However, to the best of our knowledge, this is the first study focusing on octogenarians in a medical ICU. Previous studies have shown that very old patients admitted because of medical diagnoses have a worse outcome than patients admitted due to elective or unplanned surgery.^[3,4,15,17]^ Nevertheless, the intra-ICU mortality of almost 19% as shown in the present study corresponds to the findings in other studies from surgical or mixed ICUs raging between 12% and 37%.^[3–5,17,21]^ This wide range of mortality rates can probably be explained by different ICU admission strategies, triage decisions, treatment intensities, and severity of illness in different hospitals and countries. We could show that octogenarians surviving ICU treatment were more frequently admitted due to cardiovascular diseases, especially for monitoring post PCI and after pacemaker or defibrillator implantation whereas nonsurvivors were more often admitted due to cardiac decompensation, after resuscitation or because of respiratory diseases or sepsis. Furthermore, we could demonstrate that, among medical diagnoses, the admission cause of myocardial infarction is an independent risk factor for long-term mortality whereas cardiac arrhythmia and monitoring post PCI was independently negatively correlated with the risk of mortality. Our study revealed that distinct medical admission diagnoses are much stronger predictors for mortality in octogenarians than age or SAPS II scores as other authors could demonstrate for distinct surgical diagnoses like coronary artery bypass graft (CABG) or valve surgery.^[2,17]^

There are several limitations of this study. First, it is a single center retrospective observational study. Hence the given data might not be representative even though data were similar to previously described multicenter studies. Second, there might be a selection bias because we did not record data on ICU admission and refusal rates. Admission of patients varies between institutions, physicians, and might be even dependent on ICU bed availability. Furthermore, the criteria for ICU admission for young patients might be very different from the criteria for older people making a comparison of octogenarians admitted to an ICU with a group of younger subjects debatable. A prospective study with clear admission criteria would allow such a comparison. In the present study, decision about ICU admission was done by the responsible ICU consultant. We believe that we present data in a large ICU population, describing the current practice in a real-world scenario. However, the lack of a unified admission checklist was not part of the study. Third, long-term follow-up is limited to data on survival status. Thus we have no data on cause of death, functional status, or quality of life after ICU discharge as these data were not systematically recorded in our ICU data base. Despite these limitations we believe that our data clearly show that chronological age is not the best predictor in the very old and that additional prospective multicenter studies are needed to better predict clinical needs and outcome of octogenarians.

In conclusion, octogenarians show a higher intra-ICU and long-term mortality in comparison with younger patients admitted to a medical ICU. Still, they show a considerable life expectancy after ICU admission even though they get less invasive care than younger patients. Furthermore, some admission diagnoses like myocardial infarction, cardiac arrhythmia, and monitoring post cardiac intervention are much stronger predictors for long-term survival than age or SAPS II score in the very old.

## Acknowledgments

We would like to thank Katharina Bannier and Julian Gonschorrek for their support in collecting the patients’ follow-up.
